# The Welfare of Migrants: World's Y.W.C.A. Report

**Published:** 1921-09-17

**Authors:** 


### THE PUBLIC WELL-BEING.

### THE WELFARE OF MIGRANTS.

### World's Y.W.C.A. Report.

By a SOCIAL WORKER.

It is time that an organised attack was made upon the haphazard conditions under which migration in search of better conditions from country to country at present goes on. Since the war, of course, its volume has increased, and countries to which the movements are directed continue to make more and more stringent regulations in self-protection. Such regulations, little understood in countries from which the stream flows, are for the time adding to the misery, the economic loss, the sickness, the demoralisation, the disappointment and ruin of a number of the rejected. Many of these have sold up their homes in order to finance the journey; they are thrown upon the world penniless, and though the steamship lines are bound to return them to their country of origin if the tickets were bought in that country, it is not surprising that many refuse to go back as "returned empties" to add to the trouble from which they fled. Sometimes the family is broken up by the refusal to admit certain members of it, such as an illiterate father, a feeble-minded child, or one suffering from undiscovered tuberculous trouble, or from disease contracted during the journey.

Most of these difficulties could be avoided. For some years the Emigration Committee of the National Council of Women, whose convener is Mrs. Allan Bright, has laboured for a corresponding medical standard at all ports, which would obviate the misery and waste caused by rejection on landing. Not less necessary, it may be added, is a corresponding literary test. It is absurd that this should vary so much that an immigrant may be passed in one place on his own statement, at another by his written signature, but at another properly tested by the reading of printed cards. It should surely be a detail of primary education in every country that young people should grow up understanding that illiteracy of an intending entrant to certain lands of hope means deportation, also that an intelligent adult can quickly learn to read in his own tongue. In Japan an emigrant is now only given his ticket when he has shown certificates of attendance at the emigration institute subsidised by the Government at Kobe. Here separate classes for men and women are held daily to explain the manners and customs of the Western world; its religion also, though without proselytising effort. In Italy, Jugoslavia, and some other places, classes are now also arranged.

Those mentioned in Japan are organised by the World's Young Women's Christian Association, which has now a special Migration Secretary and a Bureau for Research and Information in London (34 Baker Street, W.). This effort has grown out of a questionnaire sent round a couple of years ago to twenty-seven National Secretaries of the Y.W.C.A. Societies, of which this is a Federation. Several other countries have also appointed special secretaries, and in the report lately issued the Association urges upon all whom it can reach that the movement going on throughout the world should if rightly managed do much to make nations understand one another, and gives also a great opportunity for Christian service. It gives the results of its questionnaire in broad outlines, and a number of detailed cases, and suggests certain remedies.

To begin with, the time occupied on journeys is often far longer than is required by ordinary travellers. From Buchs to Vienna an express takes eleven hours, an emigrant train two to five days: it is often side-tracked for a time or attached to a freight train. Corridor carriages seem seldom available anywhere; water is seldom available; the poor travellers may fill bottles when stopping at a station and try to wash at station pumps. They start from home with food, but when this is exhausted the difficulty of getting more is very great; they often sleep on floors of waiting-rooms; both births and deaths occur *en route*; the plight of children who need milk is terrible, despite efforts by the Red Cross organisations. And this, let us remember, is probably merely the prelude to a trying sea-voyage. The trouble of verminous persons and their infection of others in crowded trains is most serious; proper provision of cleansing plants and of clean rooms for those who have passed through the process, more careful handling of their limited wardrobes during disinfecting treatment are needed. Robbery of emigrants, sometimes by persons professing to be travel or police agents, leads to many tragedies, and the demand is earnest for workers who will befriend in all ports those who are left stranded by shortness of money for extra expenses. As for the feeble-minded, epileptic, and insane, no country desires them, and America sternly excludes. Diagnosis at home and proper treatment there should save needless cost and grief. But if America is stern in this matter, her Immigration Law has an excellent provision by which an alien permanently resident whose family come to join him may deposit 165 dollars for the treatment in hospital on Ellis Island if any arrive ill with curable complaints.

Co-operation, education, a mutually helpful medical standard, decent arrangements on the journeys, wise and sympathetic Christian workers willing to do *en route* whatever happens to be necessary in the individual case, and finally protective action against harpies infesting the way are immediate needs. The civilised world owes gratitude to the World's Y.W.C.A. for its revelations and its suggestions. It should be added that emigration for young women alone, except as regards interchange between Great Britain and British Dominions, is not encouraged, except where definite and personal arrangements have been made.

